# Arrearages

**Published:** 1869-11

**Authors:** J. Adams Allen

**Affiliations:** 503 Michigan Avenue, Chicago


### *ARREARAGES—PLEASE NOTICE.*

ALL persons indebted to the JOURNAL for subscriptions for the present and previous years, or for advertisements in the same, are respectfully requested to remit the amount due to the undersigned or to his order only. I have advanced largely in sustaining it, at great personal inconvenience, and now that the future of the JOURNAL is secured beyond contingency, I trust that these dues, *personally my own*, may be liquidated at the earliest possible opportunity.

J. ADAMS ALLEN,

*November* 9, 1869. 503 *Michigan Avenue, Chicago.*

